# Supplementary material for: HPRNA: Predicting synergistic drug combinations for angina pectoris based on human pathway relationship network algorithm
Source: PLoS One. 2025 Feb 6;20(2):e0318368. doi: 10.1371/journal.pone.0318368 (PMC11801531; doi:10.1371/journal.pone.0318368)
Supplement: Attachment — Submitted filename: Response to reviewers.pdf [file pone.0318368.s002.pdf]

Dear Dr. Vinod and Reviewers:

Thank you for your valuable and constructive feedback on our manuscript. We truly appreciate the time and effort you have devoted to reviewing our work. In response to your comments, we have made revisions to the manuscript to address all the points raised.

Specifically, we have ensured that the manuscript fully complies with PLOS ONE's formatting guidelines, including the use of the correct LaTeX template and proper file naming conventions. Regarding the mismatch between the "Funding Information" and "Financial Disclosure" sections, I carefully compared the first submission and the revised submission interfaces. I found that the "Financial Disclosure" module was missing in the "Additional Information" section, which prevented me from modifying it. If possible, I would like to update the "Financial Disclosure" section to state: "This work was supported by the National Natural Science Foundation of China (Grant No. 12231018). The funder had no role in study design, data collection, data analysis, or preparation of the manuscript."

Additionally, we have organized the code and data to ensure they will be made publicly available upon acceptance, in accordance with PLOS ONE's open data policy. Furthermore, we have ensured that Figure 1 is properly referenced in the text, so it will be correctly linked to the corresponding content during production.

Once again, thank you for your thoughtful feedback. We hope the revisions we have made will meet the expectations for publication in PLOS ONE. Below, please find our detailed responses to the reviewers' comments.

Sincerely,
Guiying Yan

Response to Reviewer #1

Comment 1: Shouldn't the literature review encompass more contemporary studies, particularly those employing deep learning methodologies?

Response: Thank you for your suggestion. We have incorporated additional references to deep learning-based approaches in the revised manuscript. However, since our method is a mathematical model based on computational techniques, we have primarily focused on reviewing relevant work related to mathematical and computational models.

Comment 2: How does their approach stack up against machine learning or deep learning-based techniques?

Response: Thank you for this insightful question. Our approach differs significantly from machine learning and deep learning techniques, particularly when it comes to dealing with sparse labeled data. Machine learning and deep learning methods generally rely on large, labeled datasets for training and learning patterns from the data. However, our dataset is sparse, and the availability of labeled samples is limited, which can hinder the performance of these methods. In contrast, our mathematical model does not require a training phase and it directly calculates the synergy scores for drug combinations using the known drug-target and pathway relationships. This makes our

approach more effective and efficient when dealing with sparse data. Additionally, our model requires fewer computational resources compared to deep learning methods, which can be resource-intensive.

Comment 3: It would be beneficial if they could provide a detail of the score for real drug combinations.
Response: Thank you for your suggestion. We believe that the score is primarily a performance evaluation metric, and its numerical value may not hold significant practical value in isolation. As such, we did not include the detailed scores in the main text. However, we provided the scores for the top ten drug combinations in the table below for reference.

Drug A	Drug B	Score
Aliskiren	Ramipril	26489122129.84347
Morphine	Naltrexone	185766301.8451336
Propoxyphene	Prednisolone	332463.61824711866
Atorvastatin	Rosuvastatin	278093.95013719535
Phenylephrine	Pyrilamine	23284.78811070251
Pentazocine	Fimasartan	15782.652403261587
Pyrilamine	Fimasartan	15782.652403261587
Bupivacaine	Fimasartan	928.2799275187634
Aliskiren	Valsartan	325.6145388809588
Aliskiren	Losartan	325.6145388809588

Response to Reviewer #2
Comment 1: They should use more data to verify the reliability of the method.
Response: Thank you for your valuable comment. Due to limitations in available data, we are currently unable to apply our method to additional datasets. Our approach relies heavily on drug-target and pathway information, which is often not available in many publicly accessible datasets. We hope this clarifies the situation, and we will continue exploring ways to expand the applicability of our approach as more comprehensive datasets become available.

Comment 2: In order to clearly present its work, the author should add a detailed method flow chart to facilitate the reader's understanding of the process.
Response: Thank you for the suggestion. To improve the clarity of the manuscript and help readers better understand the methodology, we have added a detailed flowchart of the method in the revised manuscript. We believe this visual representation will facilitate the comprehension of our approach and provide a clearer overview of the process.

Comment 3: In the description of the method, there are some obvious expression errors, which should be carefully checked by the author.

Response: Thank you for pointing this out. We have carefully reviewed the manuscript and made corrections to the language and expression errors in the method description. The revised manuscript now reflects these improvements, and we have ensured that the wording is clearer and more accurate.

Response to Reviewer #3

Thank you for your review and valuable feedback. We have made the following revisions based on your suggestions:

Corrected the spelling error and fixed the incomplete sentence.

Added the appropriate citations and clarified the language where needed.

We appreciate your time and effort in reviewing our manuscript, and your comments have helped improve its quality. Please let us know if you have any further questions or suggestions.

Response to Reviewer #4

Thank you very much for your detailed feedback and valuable suggestions. We have carefully addressed each of your points in the revised manuscript. Below is a point-by-point response to your comments:

Comment 1: Abstract: Check spelling of 'caculate', 'investig', and 'judgment'

Response: We appreciate the reviewer's comments on the language. We have carefully reviewed the manuscript and made the necessary corrections, including fixing spelling and grammatical errors. Specific changes include:

"caculate" corrected to "calculate"

"investig" corrected to "investigate"

"judgment" corrected to "judgement".

Additionally, we have revised several sentences for clarity and readability throughout the manuscript.

Comment 2: Rephrase: "Especially for complex diseases, such as cancer and cardiovascular and cerebrovascular diseases."

Response: We have rephrased this sentence as follows: "New drug research and development faces complex processes, high economic costs, and challenges such as transient resistance, especially for complex diseases like cancer, cardiovascular diseases, and cerebrovascular disorders." This revision maintains the original meaning while improving the clarity and flow of the sentence.

Comment 3: Incomplete sentence: "There are many types of heart disease such as arrhythmia, angina pectoris."

Response: We have corrected this sentence to: "There are several types of heart disease, including arrhythmia and angina pectoris." This revision ensures that the sentence is complete and clear.

Comment 4: Which approach? "However, this approach is costly, time-consuming and poor efficiency."
Response: We have rephrased this sentence as follows: "However, these traditional experimental methods are costly, time-consuming, and have low efficiency."

Comment 5: Mention clearly which parameters are important: "Due to the time and economic cost saving, more and more people have turned ....combinations."
Response: We have revised this sentence to clarify the role of mathematical models in drug screening: "With the rapid advancement of computational technologies, various mathematical model-based methods for screening drug combinations have emerged. These computational approaches are becoming increasingly popular due to their ability to save time and reduce costs."

Comment 6: What does DREAM stand for?
Response: DREAM Challenges (dreamchallenges.org) are collaborative competitions that pose important biomedical questions to the scientific community and evaluate participants' predictions in a statistically rigorous and unbiased way, emphasizing model reproducibility and methodological transparency.

Comment 7: Authors must discuss some lines on how the models are best: 'The two best performing methods in the DREAM challenge of the drug combinations in 2014 are Genomic Residual Effect (DIGRE) and IUPUI CCBB.'
Response: We have expanded the discussion on the DREAM challenge and the best-performing models: "In the 2014 DREAM challenge on drug combinations, the DIGRE and IUPUI_CCBB models were among the best-performing methods, both relying on deterministic mathematical formulations. DIGRE effectively utilized a residual effect approach to model the complex relationships between genomic data and drug responses, providing accurate predictions based on mathematical principles. Similarly, the IUPUI_CCBB model employed a robust mathematical framework that integrated multiple biological layers, optimizing drug efficacy predictions through sophisticated mathematical modeling."

Comment 8: Poor language: 'Due to the time and economic cost saving, more and more people have turned their attention to these methods.' Consider rephrasing.
Response: We have rephrased this sentence as follows: "With the rapid advancement of computational technologies, various mathematical model-based methods for screening drug combinations have emerged. These computational approaches are becoming increasingly popular due to their ability to save time and reduce costs."

Comment 9: Authors should give a comparative remark on each model, such as DIGRE, IUPUI, NLSS, etc. mentioned in the introduction. And how the authors' model is better.
Response: We appreciate this suggestion and fully acknowledge the importance of comparing our model with existing models such as DIGRE, IUPUI, and NLSS. However, due to the limited data available on angina pectoris in our study, as well as

the differences in the datasets used by these models, a direct comparison was not feasible at this stage. Nonetheless, we plan to conduct a comparative analysis with these models once additional data is collected in the future. We will include such comparisons in a subsequent version of the study to further validate the performance of our model.

Comment 10: What is WWI and PPI?
Response: We have clarified the terms "WWI" and "PPI" as follows: "WWI refers to a method for predicting drug combinations, which is discussed in the Performance Evaluation section. PPI refers to Protein-Protein Interaction networks, which are crucial for understanding the biological interactions between proteins."

Comment 11: Better to use the word 'need' in place of 'urgency'.
Response: We have replaced the term "urgency" with "need" as suggested, to better reflect the context of the discussion.

Comment 12: Which sources were used to collect the data and how? ".... integrating data from multiple sources..."
Response: In the "Reconstruction of the drug dataset for angina pectoris" section, we have provided more detailed information about the data sources and how the data were integrated into the study.

Comment 13: Delete the paragraph "The structures of this paper.... future research as it is not required."
Response: We have deleted the paragraph "The structures of this paper..." as it was not necessary and did not contribute to the manuscript.

Comment 14: Cite suitable references for the paragraph 'Drug targets refer to the binding sites... generally important basis.'
Response: We have added relevant citations to support the statement regarding drug targets and their binding sites.

Comment 15: Write a key highlight of the algorithms discussed (Dijkstra algorithm [17], Bellman-Ford algorithm [18], and Floyd-Warshall algorithm [19]).
Response: Here are the key highlights of the algorithms discussed:

1. Dijkstra Algorithm: Efficient for finding the shortest path from a single source to all other nodes in a graph with non-negative edge weights.
2. Bellman-Ford Algorithm: Can handle graphs with negative weight edges and detect negative weight cycles.
3. Floyd-Warshall Algorithm: A dynamic programming-based algorithm for finding the shortest paths between all pairs of nodes in a graph. It works for both positive and negative edge weights and has a time complexity of O(V^3), making it suitable for dense graphs or small to medium-sized networks.

Comment 16: Useless information: 'DrugBank is a comprehensive database that...

related target information from this database.' Authors should focus on how these databases were used to procure the data in the present study.
Response: We have removed unnecessary details about DrugBank and focused on how it was used in this study to obtain drug-target information relevant to our model.

Comment 17: Expand KEGG.
Response: Thank you for your comment. KEGG stands for Kyoto Encyclopedia of Genes and Genomes. We have already included its full name in the article.

Comment 18: Figure 1 is not cited in the text. Also, the content is unclear. What do the sequences 1-3 signify? More details are required in the discussion.
Response: We have correctly cited Figure 1 in the text and clarified that the sequences 1-3 represent the steps involved in data collection. Additional explanations have been added to the discussion to provide more details about the figure.

Comment 19: Why was the value -100 chosen even if stable results were achieved at -50? Is C a unitless quantity?
Response: C is the hyperparameter of the model. The effect of choosing -50 or -100 for this parameter is essentially the same, and we randomly selected -100 for convenience. In fact, selecting -50 would not have impacted the results of our model. Additionally, we have stated that 'C' is a unitless quantity, as it is a hyperparameter in our model and does not correspond to any physical units.

Comment 20: Table 1, 2, and 3 can be combined.
Response: We believe that Tables 1 and 2, which focus on the impact of different hyperparameters on the algorithm, should remain separate from Table 3, which assesses the effects of different input data on the model's performance. We believe keeping them separate provides clearer presentation.

Comment 21: Rewrite the discussion for better clarity: "This result can be explained by the results found in the paper of Chen et al... algorithm, the results will not be much different."
Response: We have rewritten the discussion for clarity: "This result can be further explained by the findings in the study by Chen et al., which suggest that synergistic drug combinations are often associated with specific key biological pathways. These pathways are crucial in mediating the effects of drug interactions and are often central to the mechanism of synergy. In the context of our algorithm, as long as the relative distance between these key pathways remains stable throughout the computational process, the resulting predictions of drug combinations will not significantly deviate from one another. This is because the underlying biological relationships between the pathways are preserved, ensuring that the synergy predicted by our model aligns closely with the established understanding of these molecular interactions."

Comment 22: Rewrite the caption of Fig. 2 by inserting more information about the

figures.
Response: We have revised the caption for Fig. 2 to provide more detailed information about the figure's content and its relevance to the manuscript.

Comment 23: Similarly, rewrite the caption of Fig. 3 by incorporating significant discussion of the figure.
Response: We have updated the caption for Fig. 3 to include a more detailed explanation of the figure, providing context.

Comment 24: Also, include the comparison of literature models in the section 'Comparison of different inputs'. Also, discuss how the present model is better than those available in the literature.
Response: We have added several comparative models in Comparison of different algorithms. In this part, the control model remains unchanged while the input data is varied to demonstrate the accuracy and effectiveness of the data we have collected.

Comment 25: A conclusion section must be added in the manuscript that highlights the key discussion of the model described in the present paper. Also, the future prospects are too general. This information must be rewritten and provided within the conclusion section.
Response: We have added a conclusion section summarizing the key findings of our study. This section also provides more specific directions for future research, moving beyond general statements to focus on concrete future goals.

Comment 26: Authors should rewrite the manuscript by incorporating the most recent models established between 2020-2024 and submit it to a more specialized Mathematics journal.
Response: We appreciate the reviewer's comment. In our recent literature search, we found that many of the recent models for drug combination prediction (from 2020 to 2024) are based on deep learning and machine learning approaches. These methods generally require large, labeled datasets for supervised learning. However, the dataset available for our study is relatively small and does not contain sufficient labeled information to apply these techniques effectively. Moreover, given the limited scope of the available data, we have opted to use a mathematical modeling approach.

Although we have not directly compared our model with these newer machine learning or deep learning methods, we acknowledge their importance and potential. We have added relevant references to recent studies on machine learning and deep learning methods in the "Related Work" section to provide context and highlight the distinction between our approach and these newer techniques. In future work, as we gather more data, we plan to explore the integration of machine learning and deep learning approaches to further enhance drug combination predictions.

We hope the revisions meet your expectations, and we are grateful for your insightful comments. We look forward to your feedback.

Response to Reviewer #5:

Thank you for your valuable feedback and for taking the time to review our manuscript. We appreciate your constructive comments and suggestions. Please find our responses to the points you raised below:

Comment 1: Missing Latest Synergistic Drug Combinations in the Introduction

Response: We appreciate your observation regarding the outdated references in the introduction. Our approach is based on a mathematical model, and as you pointed out, many recent studies in this field have focused on machine learning and deep learning methods. In our initial submission, we inadvertently overlooked incorporating recent advancements in these areas. We have now updated the introduction to include the latest research on synergistic drug combinations, specifically focusing on deep learning and machine learning approaches. These new references and insights help contextualize our work in the current landscape of synergistic drug combination therapies.

Comment 2: Model Comparison.

Response: Thank you for pointing out the need for additional baseline comparisons. To address this concern, we have expanded our model comparison and included several additional baselines for a more comprehensive evaluation. Specifically, we have added four new methods for comparison: TarOverlap, TarDis, PathOverlap, and PathDis. These comparisons are now presented in Fig 4, and we believe this makes our model's performance more robust and convincing by providing a broader context for evaluation.

Comment 3: Conclusion Section.

Response: We apologize for the oversight regarding the conclusion section. We have now added a clear and concise conclusion to the manuscript at the end of the paper, summarizing the key findings, contributions, and future directions of our research.

We believe that these revisions significantly strengthen the manuscript, and we hope they address your concerns effectively. Once again, thank you for your helpful feedback. We look forward to your further comments.

Response to Reviewer #6:

We greatly appreciate your constructive feedback on our manuscript. We have carefully considered all of your suggestions and made the necessary revisions. Please find our detailed responses to each of your points below:

Comment 1: The introduction could benefit from a more detailed background on the challenges faced by current drug combination therapies for angina pectoris to better highlight the necessity and novelty of the HPRNA algorithm.

Response: We thank the reviewer for this valuable suggestion. In response, we have expanded the introduction to include a more detailed background on the current challenges in drug combination therapies for angina pectoris. Specifically, we discuss

the limitations of traditional approaches, such as trial-and-error methods and their inefficiency in predicting synergistic drug effects. We also highlight the importance of computational methods in overcoming these challenges, thus setting the stage for the introduction of the HPRNA algorithm as a novel solution. These additions help to better emphasize the necessity and novelty of our approach.

Comment 2: Including a separate section or a figure to visually represent the workflow or architecture of the HPRNA would aid in making the algorithmic contributions clearer, especially for readers not familiar with computational models.
Response: We appreciate this suggestion and agree that a visual representation of the HPRNA workflow would enhance clarity. In response, we have added a new figure that outlines the step-by-step workflow and key components of the HPRNA algorithm. This figure provides a clear and concise overview of how the algorithm works, which should help readers, particularly those less familiar with computational models, better understand our approach.

Comment 3: Updating and expanding the references to include recent studies that have utilized similar computational approaches in drug synergy prediction would better frame the manuscript within the current scientific landscape.
Response: Thank you for pointing this out. In response, we have updated the reference list to include several recent studies that use similar computational approaches for drug synergy prediction. This update provides a more comprehensive context for our work and aligns it with the current state of research in the field. We believe this strengthens the manuscript by demonstrating how our algorithm builds on, and contributes to, the recent advancements in drug combination therapies.

Comment 4: A few typos and grammatical errors need correction. For instance, 'caculate' in the abstract should be corrected to 'calculate.'
Response: We have carefully reviewed the manuscript and corrected the identified typographical error ("caculate" to "calculate") as well as several other minor language issues throughout the text. We believe these corrections have improved the overall clarity and readability of the manuscript.

Comment 5: The manuscript indicates that data are available from Drugbank, DCDB, and KEGG. Providing more specific details or direct links to these resources would enhance reproducibility.
Response: Thank you for this helpful comment. We would like to clarify that our data will be made publicly available upon publication.We will upload it to a publicly accessible repository to ensure transparency and reproducibility. Additionally, we have included more specific information on the data sources, including relevant links to the DrugBank, DCDB, and KEGG databases, which will allow readers to access the data directly.

We hope that the revisions we have made in response to your comments have addressed

your concerns. We greatly appreciate your feedback, and we believe that the manuscript has been significantly improved as a result. Thank you once again for your insightful suggestions.

Response to Reviewer #7:

We would like to express our sincere gratitude to the reviewer for the insightful comments and constructive feedback. We have carefully addressed each of the points raised. Please find our responses to each comment below:

Comment 1: Compare the proposed algorithm with recently developed algorithms.

Response: Thank you for pointing out the need for additional baseline comparisons. To address this concern, we have expanded our model comparison and included several additional baselines for a more comprehensive evaluation. Specifically, we have added four new methods for comparison: TarOverlap, TarDis, PathOverlap, and PathDis. These comparisons are now presented in Fig 4, and we believe this makes our model's performance more robust and convincing by providing a broader context for evaluation.

Comment 2: Choose one or more quality metrics for evaluation of proposed algorithms.

Response: Thank you for this valuable suggestion. We have used the area under the curve (AUC) from receiver operating characteristic (ROC) curves as the primary quality metric to evaluate the performance of our algorithm. A higher AUC indicates better predictive accuracy, reflecting the algorithm’s ability to distinguish between synergistic and non-synergistic drug combinations. We have included the AUC values in the results section and presented them in the corresponding figures for better clarity. This provides a clear and effective evaluation of the algorithm's performance.

Comment 3: Rewrite the conclusion section with scientific information.

Response: We agree with the reviewer that the conclusion section can be further refined. In response, we have rewritten the conclusion to focus more on the scientific significance and implications of our findings. The revised conclusion emphasizes the effectiveness of the HPRNA algorithm in predicting synergistic drug combinations for angina pectoris, discusses the strengths of pathway-based approaches, and outlines the potential for future research directions, such as extending the model to other diseases or integrating more complex biological data. We believe this revision makes the conclusion more informative and scientifically grounded.

Comment 4: Objectives of the manuscript require more specific with proposed algorithms.

Response: We appreciate the reviewer’s suggestion. We have revised the manuscript to more explicitly outline the specific goals of the study and how the proposed HPRNA algorithm addresses these objectives. The objectives now clearly state that the aim of the study is to develop a pathway-based algorithm for predicting synergistic drug combinations for angina pectoris, and to validate this approach using existing data. We also provide a clearer description of the problem the algorithm aims to solve, i.e.,

the limitations of traditional methods in identifying effective drug combinations for complex diseases like angina.

We hope that the changes made in response to your comments have improved the manuscript. We greatly appreciate your valuable feedback and believe these revisions strengthen the manuscript significantly.
